# A new solution method for wheel/rail rolling contact

**DOI:** 10.1186/s40064-016-2128-2

**Published:** 2016-04-18

**Authors:** Jian Yang, Hua Song, Lihua Fu, Meng Wang, Wei Li

**Affiliations:** School of Mechanical Engineering and Automation, University of Science and Technology, Anshan, 114051 Liaoning China; Railway Engineering Research Institute, China Academy of Railway Sciences, Beijing, 100081 China

**Keywords:** Explicit–explicit, Implicit–explicit, Wheel/rail rolling contact, Finite element model, Pre-loading

## Abstract

To solve the problem of wheel/rail rolling contact of nonlinear steady-state curving, a three-dimensional transient finite element (FE) model is developed by the explicit software ANSYS/LS-DYNA. To improve the solving speed and efficiency, an explicit–explicit order solution method is put forward based on analysis of the features of implicit and explicit algorithm. The solution method was first applied to calculate the pre-loading of wheel/rail rolling contact with explicit algorithm, and then the results became the initial conditions in solving the dynamic process of wheel/rail rolling contact with explicit algorithm as well. Simultaneously, the common implicit–explicit order solution method is used to solve the FE model. Results show that the explicit–explicit order solution method has faster operation speed and higher efficiency than the implicit–explicit order solution method while the solution accuracy is almost the same. Hence, the explicit–explicit order solution method is more suitable for the wheel/rail rolling contact model with large scale and high nonlinearity.

## Background

With constant upgrades in operating speed and axle load of trains, the interaction between wheel and rail becomes more intense, especially for the curved track which contains larger contact normal force and tangential force as well as larger creep force and creep force moment in the contact zone. Two of the most important problems in railway system are rolling contact fatigue and corrugation caused by the long-term wheel/rail rolling contact process which seriously influence the transport safety (Li et al. 2009). Hence, the wheel/rail rolling contact has been a hot issue.

For numeric simulation, the transient wheel/rail rolling contact in railway system is a very complex and time-consuming process. Reasonable solution method would reduce the computational effort and improve the solution efficiency in a certain extent. At present, there are several related solution methods that are applied to study the wheel/rail rolling contact problem (Lu et al. 2009; Zhao et al. 2013; Zhai and Huang 1991; Zhao et al. 2014; Telliskivi and Olofsson 2001; Arias-C et al. 2010; Zhai 2007; Lian 2004; Chang et al. 2010). The representative solution methods are as follows. Lu et al. (2009) applied implicit algorithm to solve the stress of wheel/rail rolling contact, but the implicit algorithm took much time when solving the problem with large scale. Zhao et al. (2013) analyzed the transient behavior of wheel/rail rolling contact at high speed by explicit algorithm, yet did not take into account the structure pre-loading. Zhuo et al. performed the simulation of larger dynamic problems of train on PCs with self-procedure programming by the Newmark explicit and predictor–corrector integration method, which greatly improved the solving speed. However, the application range of self-procedure was relatively limited for all kinds of restrictions (Zhai and Huang 1991). Zhao et al. (2014) studied the problem of wheel/rail rolling contact by the implicit–explicit order solution method, and found that the implicit analysis results would have a certain influence on the later explicit computation and the influence effect would increase as the train speed increases for the difference between implicit and explicit algorithm.

The solution methods for wheel/rail rolling contact that are based on ANSYS/LS-DYNA mainly include the explicit solution method and the implicit–explicit order solution method. The independent explicit solution method without considering the pre-loading does not tally with the actual situation, so it is not applicable for the wheel/rail rolling contact problem. The implicit–explicit order solution method is widely used to solve the rolling contact problem of railway system which needs to consider the pre-loading. However, the implicit–explicit order solution method applies implicit algorithm to define the initial pre-loading, which requires much solution time. Moreover, the solution efficiency and convergence decrease as the model nonlinearity and DOFs (degrees of freedom) increase.

To improve the solving speed and efficiency, an explicit–explicit order solution method is put forward based on analysis of the features of implicit and explicit algorithm. Results show that the explicit–explicit order solution method has faster operation speed and higher efficiency than the implicit–explicit order solution method while the accuracy of the calculations are almost the same. Hence, the explicit–explicit order solution method is more suitable for solving the wheel/rail rolling contact problem with large scale.

## The FE model of wheel/rail rolling contact

**Fig. 1:**
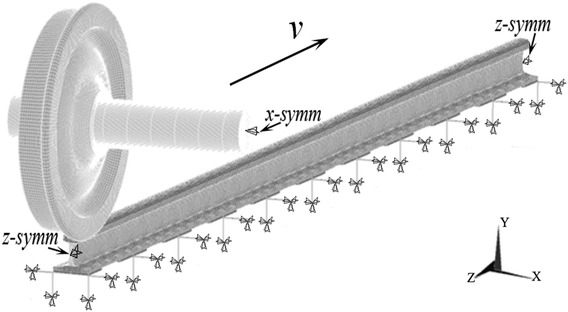
Model of wheel/rail rolling contact of nonlinear steady-state curving

The three-dimensional (3D) FE model of wheel/rail rolling contact is based on the CRH2 EMU vehicle on the Beijing–Shanghai high-speed line of China. In this model, the actual structure parameters of wheel and rail are included. The wheel profile is of the type LM_A_ and the rail is of the type CHN60 with an inclination of 1:40. A bilinear kinematic hardening elasto-plastic material model is utilized in the FE model. The application of bilinear kinematic hardening is sufficient to simulate the accumulation of plastic strain during the wheel/rail rolling contact process. And In order to reduce calculation time, the wheel is generated with rigid model. Between wheel and rail, the surface-to-surface contact algorithm is employed with a static friction coefficient of 0.2 and a dynamic friction coefficient of 0.15, provided by Wei Li (Railway Engineering Research Institute, China Academy of Railway Sciences) who is responsible for the parameter testing. The mesh near the contact surface of rail is refined to improve the contact accuracy. And the smallest length of element is about 0.85 mm which can acquire a stable and reliable solving results easily. The track system is discretized into a single-layer dynamic track model which consists of 11 groups of vertical and lateral springs and dampers. The FE model of wheel/rail rolling contact is shown in Fig. 1. The parameter values for the vertical and lateral spring are shown in Table 1 (Lian 2004), and the mechanical properties of wheel and rail, also provided by Wei Li, are shown in Table 2.

**Table 1:** Mechanical properties of wheel and rail

Properties	Value	Unit
Density (ρ)	7.83 × 10^3^	kg/m^3^
Poisson’s ratio (ν)	0.3	–
Elastic modulus (E)	2.06 × 10^5^	MPa

**Table 2:** Parameter values for lateral and vertical spring

Properties	Value	Unit
Stiffness of lateral spring (*k*_*l*_)	7.2	MN/m
Damping of lateral spring (*C*_*l*_)	108.8	kN·s/m
Stiffness of lateral spring (*k*_*v*_)	42.63	MN/m
Damping of lateral spring (*C*_*v*_)	139.8	kN·s/m

## A typical solving process of wheel/rail rolling contact

A typical wheel/rail rolling contact solving process includes two parts. Part 1: calculate the initial pre-loading (the static deformation and stress of equilibrium state) of wheel/rail rolling contact under gravity. Part 2: initialize the initial pre-loading including the deformation and stress (calculated in Part 1) and then explicitly solve the transient wheel/rail rolling contact process. Note that the initial pre-loading calculated in Part 1 is to ensure the wheelset achieves a steady-state rolling.

## Implicit and explicit algorithm

### Implicit algorithm

The Newmark method that is based on the virtual work principle is widely used in implicit solution of ANSYS. This method is of high precision and suitable for static analysis which does not consider the inertial effect. However, at each increment step, the static equilibrium equations need to be solved by iteration. The static equilibrium equation is

1 $$\hat{K}_{j} c_{j} = P_{j} - I_{j} - M_{j} \ddot{u}_{j}$$

where $\hat{K}_{j}$ is the element effective stiffness matrix, $c_{j}$ is the corrector of element displacement increment, $P_{j}$ is the element external force, $I_{j}$ is the element internal force, $M_{j}$ is the element mass matrix, $\ddot{u}_{j}$ is the element acceleration. And during each iteration, the large-scale linear equations need to be solved. In addition, very small time step is needed for the large-scale nonlinear problem such as three-dimensional transient wheel/rail rolling contact to ensure the convergence, which will take much time and computational cost. Hence, when solving the model of larger scale and higher nonlinearity, the calculation efficiency is at a relative low.

### Explicit algorithm

The explicit algorithm of ANSYS/LS-DYNA that is based on the central difference method is widely used in high-speed transient problems. There is no balance iteration and convergence problem and particularly no need to solve the element stiffness matrix. The dynamic balance condition is

2 $$M\ddot{u}=P-I$$

where *M* is the element mass matrix, $\ddot{u}$ is the element acceleration, *P* is the element external force, *I* is the element internal force. According to Eq. (2), when the increment time is *t,* the node acceleration can be defined as

3 $$\ddot{u}\mid_{t}=M^{-1}\cdot(P-I)\mid_{t}$$

In Eq. (3), the element mass matrix* M* is always symmetrical or centralized, and the node acceleration is absolutely determined by the element mass and the element force. Hence, when solving the model acceleration equations, the solution procedure is greatly simplified, which improves the whole calculation efficiency to a large extent.

### Comparison and analysis

The solution time of explicit algorithm is proportional to the number of nodes and inversely proportional to the minimum size of elements. Meanwhile, more experiences show that the solution time of implicit algorithm is roughly proportional to the number of nodes squared. So for the same model, the same element size and the same number of nodes, the explicit algorithm would take less solution time and is more efficient. Moreover, the superiority of solution time would increase quickly as the number of nodes increases relative to the implicit algorithm.

Although the implicit algorithm is provided with relatively higher solution accuracy than the explicit algorithm for the static problems (i.e. the initial pre-loading of transient wheel/rail rolling contact), the superiority of solution time of explicit algorithm would obviously exceed its shortage of solution accuracy when solving the problems with large scale and high nonlinearity. So, at the expense of a certain solution accuracy, the explicit algorithm can be used to solve the initial pre-loading of wheel/rail rolling contact instead of the implicit algorithm to reduce the solution time. Meanwhile, a large number of simulation results show that the influence of relatively small solution error of initial pre-loading can be neglected. Therefore, a new explicit–explicit order solution method is put forward to solve the transient wheel/rail rolling contact problem in this paper.

## The explicit–explicit order solution method

### Key steps of explicit–explicit order solution method

The explicit–explicit order solution method consists of three key steps. Step 1: apply the explicit algorithm to solve the initial pre-loading (the static deformation and stress of equilibrium state) of wheel/rail rolling contact only under gravity which is based on ANSYS/LS-DYNA. Step 2: import the results of deformation and stress (calculated in step 1) into the FE model of wheel/rail rolling contact to initialize the equilibrium state. Step 3: apply the explicit algorithm of ANSYS/LS-DYNA to solve the dynamic process of wheel/rail rolling contact by setting the initial velocity of wheelset and related boundary conditions. Note that the settings of boundary conditions are as follows (see Fig. 1): apply symmetric boundary conditions to the rail ends and the axle ends of wheelset, and fix all the outer ends of lateral and vertical springs and dampers.

### Time step of explicit–explicit order solution method

**Fig. 2:**
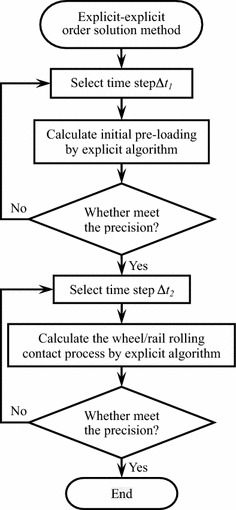
The time step determination process of the explicit–explicit order solution method

Explicit algorithm contains two kinds of errors, namely, the truncation error and the round off error. The truncation error decreases as the time step decreases. Too small time step will reduce the solution precision which is influenced by the round off error, and in some nonlinear cases (i.e. wheel/rail rolling contact) may make the results unstable. So appropriate time step is very important to the stability of wheel/rail rolling contact. The determination rule of time step is trying to select a larger time step on the premise of solution stability and accuracy. For wheel/rail rolling contact problem, the time step can be determined by the repeated numerical experiments (Zhai and Huang 1991). The determination process of time step of the explicit–explicit order solution method is shown in Fig. 2.

### Error analysis of explicit–explicit order solution method

**Fig. 3:**
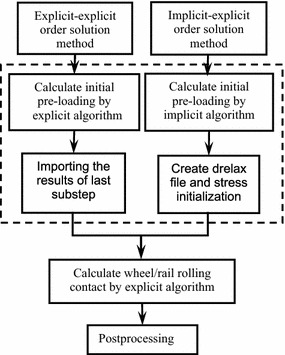
Comparison of the explicit–explicit and the implicit–explicit order solution method

The main difference between the explicit–explicit order solution method and the implicit–explicit order solution method is the type of algorithm used in solving the initial pre-loading of transient wheel/rail rolling contact. The comparison of explicit–explicit and implicit–explicit order solution method is shown in Fig. 3. Note that the differences are indicated by the dotted rectangle (see Fig. 3).

**Fig. 4:**
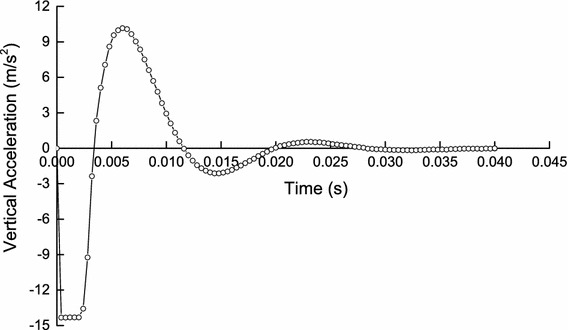
Vertical acceleration–time history curve of wheelset

**Fig. 5:**
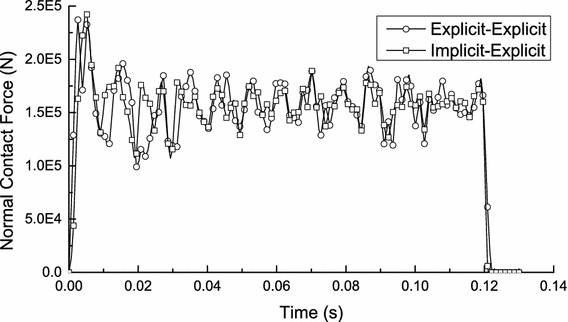
The comparison of wheel/rail contact force of two solution methods

Applying the explicit algorithm which considers the inertia effect to solve the initial pre-loading of transient wheel/rail rolling contact (a typical static analysis) will produce a certain error. However, the error can be reduced by increasing the solution time slightly (see Fig. 4). The result in Fig. 4 shows that the vertical acceleration amplitude of wheelset gradually decays to about 0 m/s^2^ during 0.02–0.04 s which is due to the effect of damping. By this time (*t* = 0.04 s), the calculation results can be used instead of the static results obtained by solving with the implicit algorithm. Meanwhile, the comparison of wheel/rail normal contact force between the explicit–explicit solution method and the implicit–explicit order solution method is illustrated in Fig. 5. The result in Fig. 5 presents that the solving accuracy of the two solution methods is almost the same. The average error of the results between the two solution methods is 6.654 % during 0.005–0.12 s, and the minimum error is 0.169 % at 0.112 s, the maximum error is 10.606 % at 0.095 s. The error between them can be reduced by increasing the solution time of pre-loading slightly as well.

In addition, the total solution time of the explicit–explicit order solution method is about one-third of the implicit–explicit order solution method (Baed on Intel(R) Core(TM) i7-3930 K CPU @ 3.20 GHz 16 G RAM), which proves that the explicit–explicit order solution method has higher calculation efficiency.

## Conclusion

According to the actual parameters, the three-dimensional transient FE model of wheel/rail rolling contact is created by ANSYS/LS-DYNA, and the single-layer track dynamic model is taken into account. Based on analysis of the features of implicit and explicit algorithm, the explicit–explicit order solution method is proposed to improve the solving speed and efficiency.

Results show that the solution accuracy of the explicit–explicit order solution method and implicit–explicit order solution method is almost the same. And the total solution time of the explicit–explicit order solution method is about one-third of the implicit–explicit order solution method. Hence, the explicit–explicit order solution method is more efficient and more suitable for solving the wheel/rail rolling contact model with large scale and high nonlinearity.
